# Social development of children with mental retardation

**DOI:** 10.4103/0972-6748.57862

**Published:** 2009

**Authors:** Indrabhushan Kumar, Amool R. Singh, S. Akhtar

**Affiliations:** Subdivisional Hospital, Pusa, Samastipur (Bihar) - 848 125, India; 1 Department of Clinical Psychology, Ranchi Institute of Neuro Psychiatry and Allied Sciences, Kanke, Ranchi - 834006, India; 2 Chief Medical Officer, Central Institute of Psychiatry, Kanke, Ranchi, Jharkhand - 834 006, India

**Keywords:** IQ, Social development, Stanford Binet intelligence scale, Vineland social maturity scale

## Abstract

**Background:**

Social development of children with mental retardation has implications for prognosis. The present study evaluated whether the social maturity scale alone can reflect on the social maturity, intellectual level and consequent adjustment in family and society of children with mental retardation.

**Materials and Methods:**

Thirty-five mentally retarded children were administered Vineland Social Maturity Scale and Stanford Binet Intelligence Scale.

**Results:**

It was found that there was significant relationship between the measures of social maturity scale and the IQ of the subjects. Further it was found that with increasing severity of retardation, social development also decreases and age does not have any effect on social development.

**Conclusion:**

Social quotient increases from profound to mild level of retardation.

Mental retardation (MR) is one of the most distressing handicaps in any society. Development of an individual with mental retardation depends on the type and extent of the underlying disorder, the associated disabilities, environmental factors, psychological factors, cognitive abilities and comorbid psychopathological conditions (Ludwik, *et al*., 2001). Social development means acquisition of the ability to behave in accordance with social expectations (Pati *et al*., 1996). Becoming socialized involves 3 processes: i) learning to behave in socially approved ways, ii) playing approved social roles and iii) development of social attitudes (Hurlock, 1967). For people with mental retardation, their eventual level of social development has implication for the degree of support needed in their literacy arrangement and their integration in the community with increasing emphasis on mainstreaming the attainment of skills in personal, domestic and community functioning. It also contributes considerably to quality of life. Thus investigation of factors that may facilitate or inhibit social development assumes particular importance.

Mentally retarded children, due to low intellectual growth, function with a limited capacity in comparison to normal children. Hence the social functioning of these children is found to be affected, and this is closely related to degree of impairment. In addition to brain pathology, there are other factors related to the malfunctioning of these children in a normal social setup. A particular environmental setup in which a child grows up is likely to play an important part in improving or deteriorating the child’s functioning in a social milieu. Shastri and Mishra (1971) assessed 56 school-going children (aged 6-13 years) with mental retardation with the help of Social Maturity Scale and found that the mentally retarded children function more in the lower level of social interaction. As the degree of impairment in terms of intelligence goes down, it is observed that the child approaches an average or satisfactory level of social functioning. They also found that the level of social development varies with the intellectual level among persons with mental retardation, or a wide range of family and environmental variables may also influence social development. Pati *et al*., (1996) designed a study to identify the effects of severity of retardation, age, type of services attended and location of services in rural/urban area on the social development of children with mental retardation using a sample of 113 subjects diagnosed as children with mental retardation. The analysis of results suggested that with increasing severity of retardation, social development also decreases. Further it was found that age, type of services and location of center do not have any effect on social development. Mayers *et al*., (1979) found that a positive relationship exists between measures of adaptive behavior and IQ or mental age. Cornbell *et al*., (1969) assessed relationship between social and cognitive functioning. For people with Down’s syndrome, the level of social functioning was found to exceed the level of cognitive functioning. Matson *et al*., (1999) designed a study to identify the effects of seizure disorders/epilepsy on psychopathology, social functioning, adaptive functioning and maladaptive behaviors using a sample of 353 people diagnosed with a seizure disorder and either severe or profound intellectual disability. People with a diagnosis of seizure disorder were found to have significantly less social and adaptive skills when compared to developmentally disabled controls with no seizure disorder diagnosis. In the light of the above investigation, the present study was designed with the following aims: (1) to find out the effects of severity of mental retardation on social development, along with possible correlation with social quotient (SQ) and IQ, which will eventually help in formulating appropriate training management and rehabilitation of mentally retarded children; and (2) to find out the relationship between age and social development.

## MATERIALS AND METHODS

### Sample

The present study was carried out on a sample of 35 mentally retarded children (mean age, 14.17 years; SD, 5.5) chosen at random from the Central Institute of Psychiatry, Kanke, Ranchi (Jharkhand). The sample included 19 males and 16 females. Children with comorbid epilepsy, sensory deficit (like impairment of vision, hearing), other psychiatric disorders and physical problems were excluded. Characteristics of the study population are given in Table 1.

**Table 1:** Characteristics of the study population

Variables	Number of subjects
Age	
Mean	14.17
SD	5.5
Range	5.02-28.02
Sex	
Male	19 (54.3)
Female	16 (45.7)
Domicile	
Urban	23 (65.7)
Rural	12 (34.3)
Monthly family income	
<2000 Rs.	10 (28.6)
2000-5000 Rs.	25 (71.4)
>5000 Rs.	0 (0.0)
Level of mental retardation	
Mild	9 (25.7)
Moderate	17 (48.6)
Severe	6 (17.1)
Profound	3 (8.6)

Figures in parentheses indicate percentage

### Tools

#### Specially designed socio-demographic data sheet

A format was developed to record the background information about the subject, like name, age, sex, level of retardation/epilepsy, etc.

#### Vineland social maturity scale (Nagpur adaptation)

The scale was originally developed by E. A. Doll in 1935, which was then adapted by Dr. A. J. Malin in the year 1965. It measures differential social capacity of an individual. It provides an estimate of social age (S.A.) and social quotient (SQ) and shows high co-relation (0.80) with intelligence. It is designed to measure social maturation in 8 social areas. The scale consists of 89 items grouped into year levels (13 age groups). It can be used for the age group of ‘below 15 years’; it means from birth to 15 years.

#### Stanford Binet intelligence scale (Hindi adaptation)

It was originally developed by Alfred Binet with the help of Simon in 1905 in France. In India its Hindi version was developed by S. K. Kulshrestha. Its 1960 revision has a range of 2 years to 22 years and 11 months of mental age scores. The single Binet L-M form is available with norms on data as recent as 1972. This form measures abilities in 7 categories: Languages, reasoning, memory, social intelligence, conceptual, numerical reasoning and visual motors. Test items are in the form of words, objects and pictures, and responses given by the testees are in the form of drawing, calculating, writing and speaking. In this revision, the intelligence is expressed in terms of standard score of intelligence, IQ.

### Procedure

Mentally retarded children were identified on the basis of International classification of disease 10^th^ revision (Diagnostic criteria for research). Informed consent was taken from the informants before eliciting relevant information, and the nature and purpose of the study were explained. All subjects who were selected for the present study were interviewed and then assessed for IQ with the help of Stanford Binet Intelligence Scale. Thereafter, Vineland Social Maturity Scale was administrated to know the level of social development of each subject.

### Analysis of data

Data has been analyzed using means, standard deviations, Kruskal-Wallis (nonparametric) one-way ANOVA test, chi-square test and Pearson correlation on social quotient.

## RESULTS AND DISCUSSION

**Figure 1:**
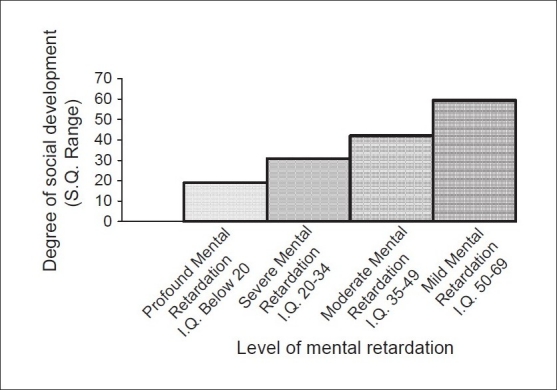
Social development in relation to various levels of mental retardation

One-way analysis of variance was carried out to find out if there was any significant difference in social development in relation to various levels of mental retardation [Table 2 and Figure 1].

**Table 2:** Difference in social development in relation to various levels of mental retardation

Level of MR	*n*	Degree of social development Mean ± SD	P*
Mild	9	59.4 ± 20.3	
Moderate	17	42.1 ± 14.4	0.002
Severe	6	30.8 ± 8.6	
Profound	3	19.0 ± 9.5	

* *P* value calculated by Kruskal-Wallis one-way ANOVA test (χ^2^ = 14.9; df = 3)

The value of ANOVA was significant at. 01 level (χ^2^ = 14.9; df = 3). This indicates that there were statistically significant differences in the social developments of children in relation to various levels of mental retardation; with degrees of social development (in terms of SQs) for mild, moderate, severe and profound retardations being 59.4, 42.1, 30.8 and 19.0, respectively, and the standard deviations being 20.3, 14.4, 8.6 and 9.5, respectively. This suggests that there are significant differences in the social development of each category of retardation. It is observed that with increasing severity of mental retardation, the level of social development decreases. The findings strongly suggest that among children with mental retardation too, the cognitive and social skills are interrelated. The intellectual development and social development go together in the same direction. Similar observations were reported by Pati *et al*., (1996).

Computed value of correlation of social quotient in different age groups was -0.17, which is not significant statistically; this may indicate the stability of social quotient with increasing age [Table 3].

**Table 3:** Correlations among age, IQ and social development

Variables	Pearson correlation coefficient	*P*
Age × social quotient (SQ)	–0.17	0.3
IQ × social quotient (SQ)	.785**	<.01

** Significant

Computed value of Pearson correlation coefficient between IQ and SQ was .785, which is significant. This may indicate relationship between IQ and SQ, i.e., relationship between intellectual capacity and social development [Table 3].

This study shows that as the level of mental retardation increases, social development decreases correspondingly. There was no impact of the age factor on the social development of mentally retarded children. Most of the people think that as the child grows, social development will be enhanced; today, he is a child; tomorrow he will be socially developed. This study will make the parents of mentally retarded children aware about the functional requirement of counseling for the mentally retarded children. If the degree of mental retardation is more, the need for special education and training will be more intense. Similarly, for those who have a lesser IQ, there will be greater requirement of special training. Most of the parents feel it is futile to spend money for the social development of severe and profound mentally retarded children; this expense will have no utility, and so why not utilize such money for normal children? This study will be helpful in making people aware about the necessity of training, managing and rehabilitating children with mental retardation. Right from the very beginning, there is an effective role for parents, teachers and other professionals in the enhancement of social skills of mentally retarded children. This study opens the path for research to determine whether there is any impact of special education and training on the social development of mentally retarded children of various age groups.

Because of time constraints and excessive workloads, trained psychologists are unable to assess the IQ, as the number of clinical psychologists throughout India is about 600-700 (Nathawat *et al*., 2001). Therefore, other methods are required for IQ assessment. Among the various techniques, social development scale is a very important method to determine one’s IQ. This social development scale is relatively easy to administer and has practical application in the assessment of IQ. It has also been found important in the management of disabled persons. SQ and IQ are highly correlated (.80) on the Stanford Binet Intelligence Scale; and the same has been found in the index study as well. Magnitude of MR is known by SQ where IQ testing is not possible. Many clinicians use social development scale in their clinics for children and adolescents as it is a valuable device for interviewing and counseling both parents and children.

## CONCLUSION

It can be concluded that the social quotient increases as level of mental retardation decreases from profound to mild. The social quotient across different age ranges does not differ significantly. Clinical psychologists who are working with underprivileged children/individuals may use the Vineland Social Maturity Scale as a rapid screening test for determining IQ and capacity for social adjustment.
